# Invasive Hemodynamic Monitoring in Acute Heart Failure and Cardiogenic Shock

**DOI:** 10.31083/RCM27034

**Published:** 2025-06-19

**Authors:** Luca Baldetti, Marcello Cosenza, Carmine Galdieri, Guglielmo Gallone, Gianluca Ricchetti, Carlo Gaspardone, Beatrice Peveri, Mario Gramegna, Lorenzo Cianfanelli, Francesco Calvo, Vittorio Pazzanese, Marina Pieri, Stefania Sacchi, Silvia Ajello, Anna Mara Scandroglio

**Affiliations:** ^1^Cardiac Intensive Care Unit, IRCCS San Raffaele Scientific Institute, 20132 Milan, Italy; ^2^Città della Salute e della Scienza, Ospedale Molinette, University of Turin, 10126 Turin, Italy; ^3^Cardiology Unit, IRCCS San Raffaele Scientific Institute, 20132 Milan, Italy; ^4^“Vita-Salute San Raffaele” University, 20132 Milan, Italy

**Keywords:** acute heart failure, hemodynamic monitoring, pulmonary artery catheter, cardiogenic shock, hemodynamics, right heart catheterization, mechanical circulatory support, intensive care

## Abstract

Invasive hemodynamic monitoring provides essential information for managing acute heart failure (AHF) and cardiogenic shock (CS) patients, aiding circulatory shock phenotyping and in individualized and hemodynamically-based therapeutic management. The hemodynamic trajectory after the initial care bundle has been provided refines prognostication and anticipates hospital outcomes. Invasive hemodynamic monitoring also tracks the clinical response to supportive measures, providing objective background for therapeutic escalation/de-escalation, facilitating titration of vasoactive/temporary mechanical circulatory support (tMCS) to achieve an optimal balance between native heart function and device assistance, and allowing for a repeated reassessment of hemodynamics during the support weaning phase. Therefore, complete hemodynamic assessment (i.e., arterial line, central venous catheter, and pulmonary artery catheter) is recommended for any patient in overt CS; however, we also provide some pragmatic clinical, imaging, and laboratory criteria to identify patients with beginning stages of CS, which could also benefit from complete invasive hemodynamic assessment. The specific hemodynamic phenotypes that can be applied in clinical practice and case-based examples of how the invasive hemodynamic phenotype can change following therapeutic actions are presented to provide pragmatic guidance on invasive hemodynamic monitoring. This review also aims to summarize the available monitoring technologies, describing the current limitations of each one and the perspective for future developments in the era of artificial intelligence. The gaps in evidence that still characterize pulmonary catheter use, i.e., lack of a robust positive randomized clinical trial in CS, are discussed, along with the wide background of non-randomized studies currently supporting its use in the CS field. The reappraisal of invasive hemodynamic monitoring, closely linked to the advent and increasing adoption of tMCS, sets the stage for greater adoption of this clinical tool in the future, as it remains a fundamental tool for the intensive care cardiologist.

## 1. Background

Acute heart failure (AHF) is a broad diagnosis encompassing a variety of different phenotypes and varying clinical severities, with cardiogenic shock (CS) at the extreme of its spectrum. CS often heralds progression to advanced heart failure and requires consideration of cardiac replacement therapies.

AHF is a leading admission diagnosis in contemporary cardiac intensive care units (CICU) [1], and invasive haemodynamics are obtained in approximately 30–40% of CICU admissions [2]. AHF includes any acute cardiac event leading to low cardiac output and/or pulmonary or systemic congestion [3]. The pathophysiology and outcomes differ significantly between acute myocardial infarction (AMI)-related AHF, acute decompensated heart failure (ADHF), and de novo non-AMI related AHF, warranting individualized treatments [4, 5, 6, 7, 8]. Subsequently, prognosis of AHF is considerably variable, depending on patient pre-existing comorbidities, the aetiology of AHF and the clinical severity upon presentation. Nevertheless, there is general consensus that clinical and hemodynamic trajectory during AHF hospitalization help to identify the leading AHF phenotype, predict overall prognosis across different aetiologies and guide therapeutic measures [9, 10, 11, 12, 13].

This review aims to summarize the available technologies and evidence on hemodynamic monitoring in AHF, CS, and advanced heart failure, providing practical recommendations for its implementation.

## 2. Invasive Haemodynamic Monitoring

### 2.1 General Principles of Invasive Monitoring

In general, invasive monitoring requires fluid-filled catheters, mandating proper levelling, zeroing, and damping to ensure accurate pressure readings. The intravascular line is connected to a pressure transducer with an automatic flushing system (i.e., a pressure bag providing infusion of pressurized saline into the pressure line). Vascular pressure fluctuations cause pulsations in the saline column, displacing the electrical manometer’s diaphragm, which contains a strain gauge based on the Wheatstone bridge principle. This deformation changes resistance, which is electronically detected and used to create a waveform through Fourier analysis. For accurate calibration, the transducer, tubing, and flush solution must be correctly assembled, and air bubbles eliminated. The transducer should be positioned at the level of the patient’s right atrium [14, 15, 16]. As a rule of thumb, this is identified by a mid-axillary line and 5 cm below sternal Louis’ angle [17]. Identification of this level in obese patients may be challenging, and may be deeper than usual.

**Fig. 1.**
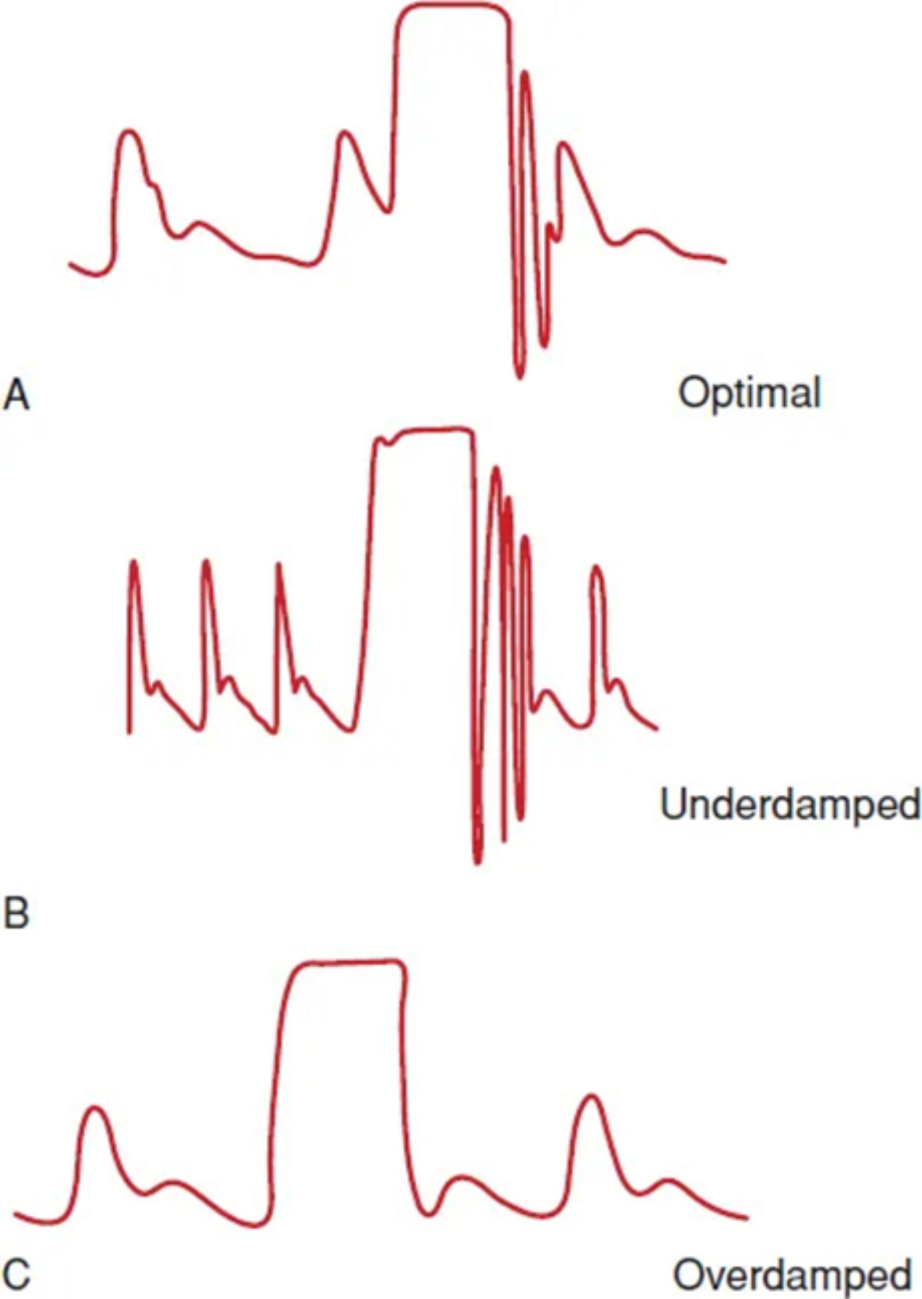
**Common pitfalls in invasive hemodynamic monitoring and pressure waveform analysis**. (A) An optimally damped pressure line: the waveform responds with 1–2 oscillations after line flushing. This ensures accurate estimation of pressure values. (B) An underdamped pressure line: the waveform responds with >2 oscillations after line flushing, leading to systolic pressure overestimation and diastolic pressure underestimation (mean pressure value is usually unaffected). This suggests insufficient density of the line filling fluid: changing the fluid with more viscous solutions (e.g., saline over glucose) should be considered. (C) An overdamped pressure line: the waveform responds with no oscillations after line flushing, leading to systolic pressure underestimation and diastolic pressure overestimation (mean pressure value is usually unaffected). This suggests obstruction of the pressure line: changing catheter position, repeated manual flushing with a syringe and deairing of the line should be considered to fix this problem.

The system must be set to “off to patient, open to air” with atmospheric pressure zeroed by pressing the “zero” button. If the patient’s position changes, the transducer height should be adjusted accordingly. Improper calibration can lead to inaccurate pressure measurements. A snap flush test generates a square wave to assess system oscillations: one oscillation is optimal, two or more indicate an underdamped system, while no oscillations suggest an overdamped system with a slow response; both these pitfalls may lead to inaccurate pressure estimation and should be corrected (Fig. 1). A summary of currently available methods for invasive hemodynamic monitoring, and their best clinical indications, is reported in Table 1.

**Table 1.** **Summary of available technology for invasive hemodynamic monitoring in acute heart failure**.

	Advantages	Disadvantages	Appropriate clinical settings
Arterial line	● Continuous invasive BP measurement	● Risk of arterial occlusion	● Respiratory failure
	● Frequent arterial blood sampling	● Difficult access in certain patients (spasm; obesity)	● Invasive mechanical ventilation
	● Analysis of the waveform (identify specific pathologies)	● Risk of retroperitoneal hematoma (femoral access)	● CS
	● Radial artery easy to cannulate (superficial)		● Ongoing vasoactive therapies
			● tMCS
Central venous catheter	● Long term access	● Complications during insertion (vascular injury, pneumothorax for internal jugular/subclavian vein)	● CS
	● Safe administration of vasoactive drugs		● Ongoing vasoactive therapies
	● Infusion of large volumes (blood products)	● Risk of infections	● tMCS
	● Assessment of CVP and SVcO_2_	● Thrombosis risk	
Flotrac-Vigileo	● Easy setup and use	● Accuracy limitations in certain conditions (arrhythmias, low cardiac output states, or significant changes in vascular tone)	● Respiratory failure
	● Connected to arterial line		● Invasive mechanical ventilation
	● No calibration required	● Cost	● Sedated patients
	● Dynamic parameters for fluid responsiveness	● Dependence on arterial waveform quality	● AHF without overt CS
	● Continuous monitoring	● Limited use in spontaneous breathing patients	
PiCCO	● Simple	● Additional arterial line required	● Respiratory failure
	● Comprehensive data (can be used to assess fluid responsiveness and extravascular lung water)	● Calibration needed	● Invasive mechanical ventilation
		● Cost	● Sedated patients
	● Continuous monitoring	● Operator dependency	● AHF without overt CS
Pulmonary artery catheter	● Gold standard	● Complications during insertion (vascular injury, pneumothorax for internal jugular/subclavian vein; pulmonary hemorrhage)	● CS
	● Continuous monitoring		● Mixed shock
	● Provides full hemodynamic assessment	● Risk of infections	● Ongoing vasoactive therapies
		● Thrombosis risk	● tMCS

Legend: BP, blood pressure; CS, cardiogenic shock; CVP, central venous pressure; SvcO_2_, central venous oxygen saturation; tMCS, temporary mechanical circulatory support; AHF, acute heart failure.

### 2.2 Arterial Blood Pressure Invasive Monitoring

Continuous arterial blood pressure (ABP) monitoring is critical for patients in CICUs with AHF or CS [18]. This is commonly achieved through radial artery cannulation due to its ease of access and low risk of complications, though the brachial and femoral arteries can also be used. ABP monitoring is especially indicated for patients with labile blood pressure, anticipated hemodynamic instability, patients in overt CS, requiring vasoactive drugs and/or temporary mechanical circulatory support (tMCS), patients with respiratory failure, or those with morbid obesity due to inaccurate non-invasive ABP measurements.

ABP lines are useful for titrating vasoactive drugs, analysing arterial blood gases, adjusting ventilation settings in mechanically ventilated patients, and allowing frequent blood sampling. Moreover, specific waveform morphologies can provide diagnostic clues (e.g., slow rising waves suggest aortic stenosis, pulsus alternans may indicate severe left ventricular (LV) failure, pulse pressure variation suggests hypovolemia and can predict fluid responsiveness).

### 2.3 Central Venous Catheter

Central venous access (CVC) is essential in critically ill patients. It enables measurement of central venous oxygen saturation (SvcO_2_), central venous pressure (CVP), and facilitates frequent blood sampling, as well as multiple drugs administration, including inotropes, vasopressors, sedatives, electrolytes, antibiotics and parenteral nutrition as needed. CICU patients with unstable hemodynamics, or those on intravenous inotropic agents and/or tMCS, as well as those with anticipated requirement of multiple continuous infusions should receive CVC insertion.

### 2.4 Pulse-contour Analysis Monitoring Systems

Peripheral arterial waveform analysis serves as the foundation for advanced hemodynamic monitoring systems, such as the PiCCO and FloTrac systems, which provide cardiac output (CO) estimates. The PiCCO system (Pulsion Medical Systems, Munich, Germany) uses a 2-element Windkessel model to calculate CO, stroke volume (SV), and arterial pressure waveform by analysing vessel compliance during systole and diastole. PiCCO combines transpulmonary thermodilution with waveform analysis for pressure readings [19].

The FloTrac system (Edwards Lifesciences, Irvine, California) offers continuous CO measurement by using pulse rate and SV without requiring recalibration. It continuously updates hemodynamic parameters such as CO, cardiac index (CI), stroke volume variation (SVV), and stroke volume index (SVi). Compared to PiCCO and pulmonary artery catheters (PAC), FloTrac provides similar CO readings, making it a suitable, less invasive alternative. The main advantage of FloTrac is its ability to connect to a standard peripheral arterial catheter, and when combined with the Edwards HemoSphere monitor, it displays CO, SV, and SVV [20].

Both these methods are limited by the lack of information on pulmonary circulation and by the poor accuracy in CO and SV estimation when tMCS devices that alter arterial waveform morphology are ongoing.

### 2.5 Pulmonary Artery Catheter

Advanced invasive hemodynamic monitoring requires right heart catheterization (RHC) with the PAC and is frequently necessary at various stages throughout the whole trajectory of heart failure (HF), encompassing both the acute phase of hospitalization and the chronic management setting. The integration of advanced hemodynamic monitoring into the management of HF patients ensures precise, data-driven decision-making, enhancing the ability to tailor therapies to the individual patient’s hemodynamic profile, especially in case of symptoms refractory to medical therapies or disproportionate to objective data. Moreover, invasive hemodynamic data are mandatory to guide clinical decision making for left ventricular assist device (LVAD) therapy and heart transplantation in more advanced stages. Basically, vascular access for RHC is usually obtained via the internal jugular, subclavian or femoral vein under safe ultrasound guidance. Right jugular vein is the preferred vascular access for bedside catheterization because the balloon-tipped catheter is more naturally directed toward the pulmonary artery without the need for fluoroscopy [21]. The PAC is then advanced through the right heart and up to the pulmonary artery to obtain CVP, right ventricular (RV) pressure, pulmonary artery pressures (PAP) (systolic, diastolic, mean), and pulmonary artery wedge pressure (PAWP). Ideally, pressure should be recorded at end-expiration, i.e., at the functional residual capacity, to avoid the impact of intrathoracic and pleural pressure swings, irrespective to the mode of ventilation (spontaneous vs. positive-pressure ventilation) [21]. Significant respiratory variations of intrathoracic pressure can be observed in patients with chronic lung disease and severe obesity, in this case average values over the entire respiratory cycle may better approximate intravascular pressures [22]. CO can be calculated using either direct methods (direct Fick and thermodilution) or the indirect Fick method and divided by body surface area (BSA) to obtain CI [21]. The direct Fick method for CO determination is assumed as the gold standard technique but requires direct measurement of the whole-body oxygen consumption (VO_2_) and is not often available, especially in the CICU setting [21]. This limitation has led to the development of indirect Fick method, that combines direct measurement of mixed venous saturation (SvO_2_) from the pulmonary artery (PA), arterial saturation (SaO_2_) from arterial blood sampling and haemoglobin concentration with the VO_2_ values estimated based on nomograms; however these nomograms assume a “normal” physiology and may not be accurate in AHF and CS patients. Currently, thermodilution-based CO is the preferred method in the CICU department and when direct VO_2_ measurements cannot be obtained. Noteworthy, thermodilution CO is unreliable in the setting intracardiac shunt and may be affected—on a minor extent—also by severe tricuspid regurgitation [21, 23]. In addition to direct haemodynamic parameters, several derived parameters can be obtained from PAC [24] (a complete summary is reported in Table 2 (Ref. [12, 24, 25, 26, 27, 28, 29, 30, 31, 32, 33, 34, 35, 36, 37, 38])). Among derived indexes, admission cardiac power output (CPO) and cardiac power index (CPI) have been shown to be strongly associated with in-hospital mortality in CS patients and can be set as hemodynamic goals to monitor treatment effectiveness. CPO is typically expressed in Watts by dividing the product of mean arterial pressure (MAP) and CO by 451 and provides a measure of combined (left and right) ventricular power [25, 26, 27, 28, 29, 30].

**Table 2.** **Summary of available technology for invasive hemodynamic monitoring in acute heart failure and cardiogenic shock**.

Hemodynamic index	Calculation	Clinical meaning	Reference range
Direct indexes			
Mean arterial pressure (MAP)	-	Systemic perfusion	70–105 mmHg
Pulmonary artery wedge pressure (PAWP)	-	Surrogate for LVEDP, surrogate for LA pressure	6–12 mmHg
			<18 mmHg (according to CSWG) [24]
Systolic pulmonary artery pressure (PAPs)	-	Pulmonary congestion; RV afterload	15–25 mmHg
Diastolic pulmonary artery pressure (PAPd)	-	Pulmonary congestion; RV afterload	8–15 mmHg
Mean pulmonary artery pressure (PAPm)	-	Pulmonary congestion; RV afterload	10–20 mmHg
Right atrial pressure (RAP)	-	RV chamber function	2–6 mmHg
			<12 mmHg (according to CSWG) [24]
Cardiac output (CO)	-	Systemic perfusion	4–8 L/min
Mixed venous oxygen saturation (SvO_2_)	-	Systemic perfusion	65–75%
Indirect indexes			
Cardiac index (CI)	CI = CO/BSA	Systemic perfusion	2.5–4.0 L/min/m^2^
Stroke volume (SV)	SV = CO/HR × 1000	Systemic perfusion	60–100 mL
Stroke volume index (SVi)	SV = CI/HR × 1000	Systemic perfusion	33–47 mL/m^2^
Cardiac power output (CPO)	CPO = (CO × MAP)/451	Global cardiac power (LV + RV)	≥1.0 W (healty individuals) [26]
			>0.53 W (AMI-CS) [29]
			≥0.60 W (AMI-CS) [28]
Cardiac power index (CPI)	CPO = (CI × MAP)/451	Global cardiac performance (LV + RV)	>0.32 W/m^2^ (AMI-CS) [25]
RAP-corrected CPO (CPO-RAP)	CPO-RAP = [CO × (MAP–RAP)]/451	Global cardiac performance (LV + RV)	≥0.66 W (HF) [27]
RAP-corrected CPI (CPI-RAP)	CPI-RAP = [CI × (MAP–RAP)]/451	Global cardiac performance (LV + RV)	>0.28 W/m^2^ (CS, general) [30]
LV stroke work (LVSW)	LVSW = SV × (MAP–PAWP) × 0.0136	LV chamber function	58–104 cJ
LV stroke work index (LVSWi)	LVSWi = SVi × (MAP–PAWP) × 0.0136	LV chamber function	50–62 cJ/m^2^
RV stroke work (RVSW)	RVSW = SV × (PAPm–RAP) × 0.0136	RV chamber function	8–16 cJ
RV stroke work index (RVSWi)	RVSWi = SVi × (PAPm–RAP) × 0.0136	RV chamber function	5–10 cJ/m^2^
Systemic vascular resistances (SVR)	SVR = (MAP–RAP)/CO	Peripheral vessels tone	10–15 WU
Systemic vascular resistances index (SVRi)	SVRi = (MAP–RAP)/CI	Peripheral vessels tone	25–30 WU*m^2^
Pulmonary vascular resistances (PVR)	PVR = (PAPm–PAWP)/CO	Pulmonary vessels tone	≤2 WU
Pulmonary vascular resistances index (PVRi)	PVRi = (PAPm–PAWP)/CI	Pulmonary vessels tone	3.2–3.5 WU*m^2^
Transpulmonary gradient (TPG)	TPG = PAPm–PAWP	Combined post-capillary hypertension	<12 mmHg
Diastolic pulmonary gradient (DPG)	DPG = PAPd–PAWP	Combined post-capillary hypertension	<7 mmHg
Arterial pulsatility index (API)	API = (SAP–DAP)/PAWP	LV chamber function	>2.9 (HF) [31]
RAP/PAWP	-	RV chamber function	≤0.86 (AMI-CS) [32]
			≤0.63 (LVAD implant) [33]
Pulmonary pulsatility index (PAPi)	PAPi = (PAPs–PAPd)/RAP	RV chamber function	≥0.9 (AMI-CS) [34]
			≥1.85–2.00 (LVAD implant) [35, 36]
			≥3.65 (advanced HF) [37]
Pulmonary elastance (PaE)	PaE = PAPs/SV	RV chamber afterload	≤0.85 mmHg/mL (HF-CS) [12]
Pulmonary compliance (PaC)	PaC = SV/(PAPs–PAPd)	RV chamber afterload	Not definite
Veno-arterial pCO_2_ gap	P(a-v)CO_2_ gap = PvCO_2_–PaCO_2_	Microvascular circulatory function	≤6 mmHg [38]

Legend: AMI, acute myocardial infarction; BSA, body surface area; CS, cardiogenic shock; CSWG, Cardiogenic Shock Working Group; HF, heart failure; LA, left atrial; LVAD, left ventricular assist device; LVEDP, left ventricular end-diastolic pressure; RV, right ventricular; SAP, systolic arterial pressure; CI, cardiac index; CO, cardiac output; DAP, diastolic arterial pressure; HR, heart rate; LV, left ventricular.

### 2.6 Artificial Intelligence

The amount of data generated by the instantaneous sampling obtained with the invasive monitoring systems provide an ideal substrate for artificial intelligence (AI) analysis. Dedicated, AI algorithms have been developed and integrated with hemodynamic monitoring platforms to predict adverse events—e.g., systemic hypotension—and allow for pre-emptive interventions. For example, the proprietary Edwards Lifesciences Hypotension Prediction Index (HPI) leverages on a dedicated arterial waveform sensor (Flotrac IQ) and a monitor (HemoSphere) to warn the clinician 5–15 minutes prior to hypotensive events [39]. This also system classifies hypotension events are related to impaired preload, afterload, and contractility, allowing for tailored upstream strategies. The HPI system, coupled with a pre-specified treatment protocol, was able to reduce intraoperative hypotension episode during non-cardiac surgery [40]. Similarly, application of AI machine learning techniques to large datasets combining hemodynamic, metabolic, and clinical information yielded accurate tools to predict CS when applied to at-risk patients [41], and to cluster different phenotypes within the CS population [42]. However, implementation of these protocol in the AHF patients is still limited, as they rely also on non-hemodynamic variables, they are not incorporated in a monitoring platform, and were generated from potentially not enough granular data [43]. In addition, specific arterial waveform alterations induced by tMCS could dramatically mislead algorithms based on waveform analysis, like the HPI.

## 3. Invasive Haemodynamic Monitoring in Acute Heart Failure at Risk of CS

During the past decades, both observational studies and clinical trial have questioned the utility of a routine bedside invasive haemodynamic monitoring with PAC among a wide range of critically ill patients due to neutral or negative impact on clinical outcomes, leading to a notable decline in its use [44, 45, 46, 47]. However, these studies suffered from several limitations, including patient selection and lack of standardized protocols in therapeutic adjustment in response to information provided by the PAC [48]. In the setting of AHF not yet complicated by CS, the challenge lies in understanding the severity of the clinical picture and the true risk of in-hospital worsening and which patient could benefit from PAC insertion.

The landmark Evaluation Study of Congestive Heart Failure and Pulmonary Artery Catheterization Effectiveness (ESCAPE) trial failed to demonstrate that, among patients hospitalized for ADHF, a routine use of PAC to adjust decongestive and vasoactive therapies was superior to a therapeutic approach guided by clinical assessment alone, with similar results in terms of length of index hospitalization and mortality and higher incidence of adverse events in the PAC group [49]. However, ADHF patients often linger some time on a “beginning” CS state (i.e., CS stage B according to the Society for Cardiovascular Angiography and Interventions (SCAI) classification, see below) and >50% of admission SCAI B patients worsen to more severe shock states at 24 hours [50]. In addition, ADHF patients with long-standing cardiac dysfunction exhibit the highest tolerance to low CO, so that even a severe hemodynamic compromise may be masked by falsely reassuring blood pressure and normal lactate as a result of chronically and disproportionally elevated systemic vascular resistances (SVR), pre-existing hypertension, and higher O_2_ extraction from end-organs [51, 52]: this may result in under-recognition and undertreatment of hypoperfusion. Indeed, reduced SvO_2_—rather than serum lactate—may be a better gauge of end-organ perfusion in this group. Therefore, hemodynamic indexes obtained with PAC offer an earlier window for CS diagnosis and intervention. In these patients PAC insertion may in fact unveil a profound low flow state and trigger therapeutic changes in up to 70% of the cases [52], and is associated to higher use of downstream advanced heart failure therapies [53]. These patients may be amenable to early supportive vasoactive therapies (e.g., sodium nitroprusside, nitrates) or even intra-aortic balloon pump (IABP) therapy [6]. Indeed, IABP, which significantly reduces LV afterload but only modestly increases CO, may be most effective in clinical situations where there is a disproportionate increase in afterload without severe hemodynamic compromise like in the SCAI B ADHF setting. Several hemodynamic indexes identify patients who would dramatically benefit from IABP in ADHF, i.e., elevated SVR, isolated LV or biventricular dysfunction, pulmonary circulation congestion, inadequate response to initial diuretic therapy and regular non-tachycardic heart rhythm. In addition, patients with ADHF may feature variable degrees of concomitant functional mitral regurgitation (MR), of moderate or severe grade in approximately 60% of the cases [54]. Modulation of MR severity (by either pharmacologic vasodilators or IABP) may easily be quantified by looking at the PAWP waveform [55], where large reductions of previously prominent v-waves indicate a good response to vasodilatation, thus also helping in choosing between goal-directed medical therapy (GDMT) intensification or MR interventional correction. Finally, PAC may also confirm the efficacy of transitioning from i.v. to oral vasodilator therapy [56, 57].

Identification of ADHF patients who benefit from PAC is challenging but some simple clinical variables may suggest an underlying severe hemodynamic derangement: sinus tachycardia; serum lactate ≥2 mmol/L; frequent ventricular arrhythmias; hepatorenal injury on blood tests; SvcO_2_ <60% or a veno-arterial pCO_2_ gap >6 mmHg [38]. Bedside, noninvasive echodynamic assessment can also trigger invasive hemodynamic monitoring based on the following markers: reduced LV stroke volume (left ventricular outflow tract velocity-time integral, left ventricular outflow tract (LVOT) velocity-time integral (VTI) <9 cm), RV dysfunction (tricuspid annular plane systolic excursion <17 mm—tricuspid annulus s’ velocity at tissue Doppler <9 cm/s), severe increase in RV afterload (systolic PAP >50 mmHg, and severe estimated systemic congestion (CVP >15 mmHg) [58]. Presence of one or—more commonly—more of these criteria should prompt consideration of PAC insertion. This also underscores the pivotal role of echocardiography in identifying candidates for PAC insertion: echocardiography is indeed a powerful and reliable tool to non-invasively estimate hemodynamics and should always be performed as a first-line assessment in the AHF patient.

Hemodynamic compromise may—on the opposite—be more obvious in AMI-related AHF, as these patients lack chronic compensatory mechanisms. Patients with extensive antero-lateral AMI, with severe depression of the LV function, and with mechanical complications are at significant risk of CS. These criteria, along with elevated serum lactate, sinus tachycardia, acute pulmonary edema at presentation, recurrent ventricular arrhythmias should prompt PAC use consideration for subsequent management [59]. At the time of percutaneous coronary intervention (PCI), direct measurement of left ventricular end-diastolic pressure (LVEDP) with the use of a pig-tail catheter can aid in identifying patients who may benefit from LV micro-axial flow pump (mAFP) unloading.

**Fig. 2.**
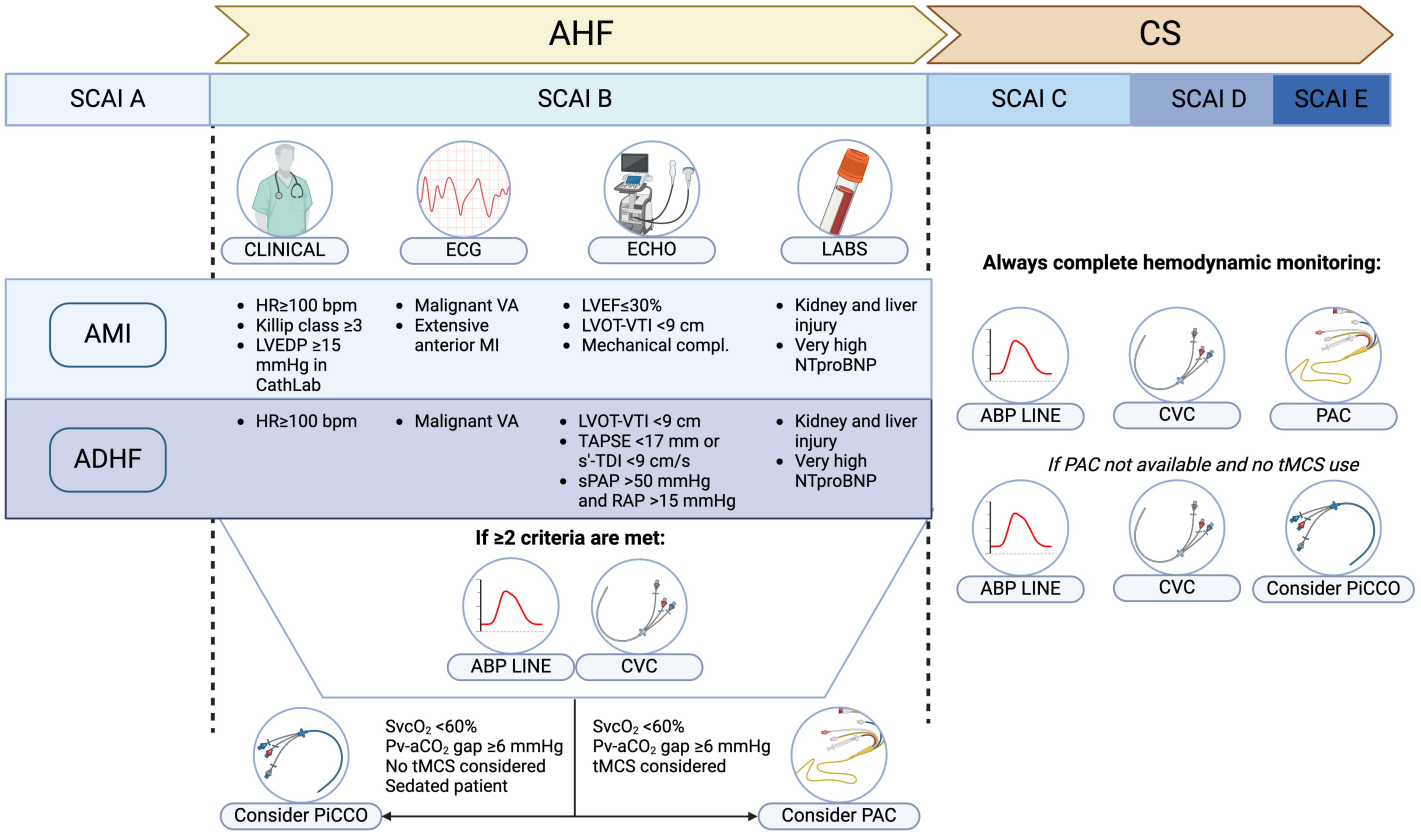
**Pragmatic algorithm for patient and hemodynamic monitoring selection based on the AHF/CS etiology**. Select criteria that warrant consideration for hemodynamic monitoring are summarized, based on the CS etiology. In case of AHF and beginning CS (SCAI B), presence of two or more of the proposed criteria warrant insertion of ABP line and CVC; if poor SvCO_2_ or high Pv-aCO_2_ gap are measured on the central venous blood sample, PiCCO or PAC insertion should also be evaluated. Notably, PiCCO would not be accurate if tMCS are instituted. In case of over CS (SCAI C or higher) a complete hemodynamic monitoring with ABP line, CVC and PAC is warranted. If PAC has a limited availability, PiCCO offers an alternative, provided that no tMCS are used. ABP, arterial blood pressure; AMI, acute myocardial infarction; ADHF, acute decompensated heart failure; CS, cardiogenic shock; CVC, central venous catheter; HR, heart rate; LVEDP, left ventricular end-diastolic pressure; LVOT, left ventricular outflow tract; PAC, pulmonary artery catheter; Pv-aCO_2_ gap, veno-arterial CO_2_ pressure gap; RAP, right atrial pressure; SCAI, Society for Cardiovascular Angiography & Interventions; s’-TDI, s’ wave on tissue Doppler imaging; sPAP, systolic pulmonary artery pressure; SvcO_2_, central venous oxygen saturation; TAPSE, tricuspid annular plane systolic excursion; tMCS, temporary mechanical circulatory support; VA, ventricular arrhythmia; VTI, velocity-time integral; AHF, acute heart failure; CS, cardiogenic shock; HR, heart rate; ECG, electrocardiogram; ECHO, echocardiography; LABS, laboratory tests; LVEF, left ventricular ejection fraction; VA, ventricular arrhythmias. Fig. 2 was created with Biorender.com.

In general, regardless of AHF etiology, any patient who fails to stabilize with the initial therapeutic measures should be considered for PAC insertions and invasive hemodynamic assessment. In addition, the specific limitations of each monitoring device mentioned in Table 1, should be considered for optimal tool selection. A pragmatic algorithm to identify the best monitoring configuration within the AHF and CS spectrum is reported in Fig. 2.

## 4. Invasive Haemodynamic Monitoring in Overt CS

Patients with CS have been conventionally excluded from clinical trials on PAC use. Recently, several observational studies evaluating association between PAC use and short-term outcomes among patients with cardiogenic shock have been released [53, 60, 61, 62, 63, 64]. Notably, hospital practice still substantially accounts for the variability observed in PAC adoption [65]. Hernandez *et al*. [63] conducted a large registry-based study collecting retrospective data with the use of the National Inpatient Sample database (NIS) in the US from 2004 to 2014, including more than 9 million of patients with a primary diagnosis of HF or who developed CS during the index hospitalization. The study demonstrated a higher mortality among patients with HF receiving PAC (9.9% vs. 3.3%), although the excess of mortality declined over time during the years of the study. Paradoxically, the use of PAC in the setting of CS was associated with improved outcomes (in-hospital mortality 35.1% in the PAC group vs. no-PAC 39.2%, OR 0.91; *p* < 0.001) [55]. Noteworthy, the increasing use of tMCS for CS represents an expanding indication for PAC [59], and tMCS use explains much of the variability observed in PAC adoption [66]. O’Neill *et al*. [62] performed a sub-analysis of the Impella IQ US registry including patients admitted with a diagnosis of AMI-CS and receiving tMCS with the Impella devices. In this study, the use of PAC for haemodynamic monitoring was found to be associated with better survival [62]. More recently, Garan *et al*. [64] collected retrospective data from the first eight sites contributing to the Cardiogenic Shock Working Group (CSWG) in the US from 2016 to 2019. The main cause for CS was ADHF, followed by AMI-CS [64]. The Authors found that early (prior to tMCS initiation) and complete invasive haemodynamic profiling with PAC was associated with lower in-hospital mortality as compared to having incomplete or no PAC assessment, across each SCAI stage sub-cohort, even after accounting for potentially confounding factors, and this difference was more pronounced among patients with greater degrees of haemodynamic compromise (SCAI D and E patients). This was true both for the AMI-CS and ADHF-CS cohorts. The currently ongoing Pulmonary Artery Catheter in Cardiogenic Shock Trial (PACCS trial; NCT05485376) will test whether PAC use in ADHF-CS would lead to lower hospital mortality. However, given the lack of high-quality evidence current guidelines do not support a systematic use of advanced haemodynamic monitoring in CS, limiting the use of PAC for the most severe cases and those who fail to respond to initial therapy or in cases of diagnostic or therapeutic uncertainty [18, 67].

**Fig. 3.**
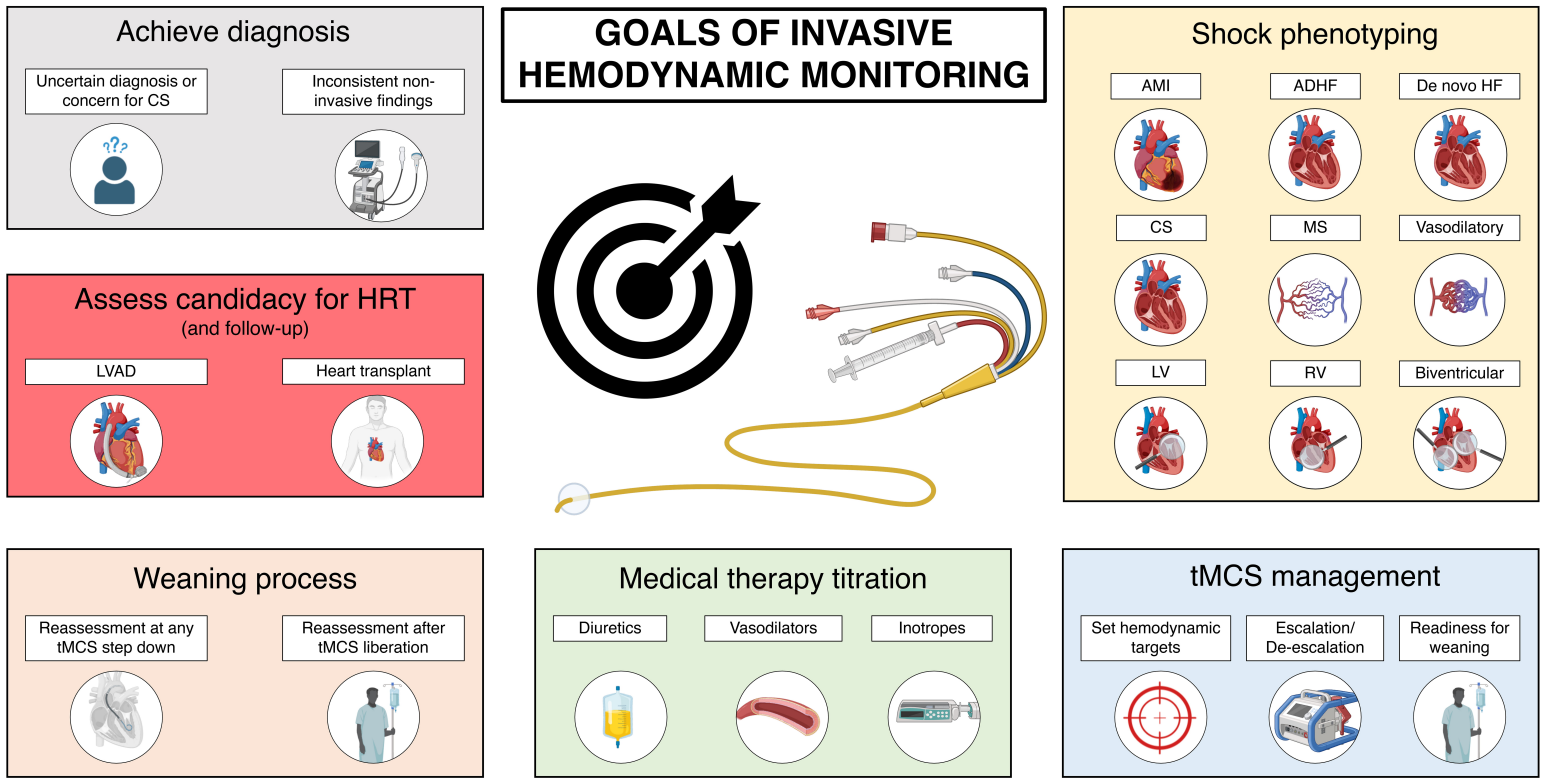
**The spectrum of goals of invasive hemodynamic monitoring in the AHF patient**. AMI, acute myocardial infarction; ADHF, acute decompensated heart failure; CS, cardiogenic shock; LV, left ventricular; LVAD, left ventricular assist device; MS, mixed shock; RV, right ventricular; tMCS, temporary mechanical circulatory support; HRT, heart replacement therapies; HF, heart failure.

Any patient presenting in overt CS should receive an arterial pressure line, and a central venous catheter and the addition of PAC should be strongly considered (Fig. 2). Indeed, the SCAI/Heart Failure Society of America 2017 expert consensus document recommends invasive haemodynamic monitoring and PAC-derived data for several purposes including (1) timely diagnosis and classification of CS based on the haemodynamic profile, (2) choice and management of supportive measures including pharmacological interventions and haemodynamic-based tMCS selection, (3) haemodynamic-based patient management during tMCS, (4) escalation of tMCS according to haemodynamic data, (5) weaning and eventually withdrawal of pharmacological and tMCS in patients with myocardial recovery and, (6) assessment for candidacy to advanced HF therapies, including durable tMCS and heart transplantation, in those who fail to recover from myocardial injury [67]. The many goals of invasive hemodynamic monitoring are summarized in Fig. 3.

### 4.1 Haemodynamic Classification of CS

Since the ground-breaking research by Forrester *et al*. [68] on phenotyping of AMI-CS patients, the role of medical treatment guided by the patient’s hemodynamic profile has been incorporated into diagnostic and therapeutic algorithms of CS. This study resulted in the creation of the renowned Forrester classification [68]. This classification links PAWP to CI to categorize patients according to their congestion (dry vs. wet) and perfusion status (cold vs. warm), with significant prognostic and therapeutic implications.Historically, patients with CS were categorized as having a low CI ≤2.2 L/min/m^2^ (cold) and a high PAWP ≥18 mmHg (wet) leading to the classic “cold and wet” profile, which still represents the most frequent CS phenotype in the AMI-CS group accounting for nearly two-thirds in the SHOCK trial population and the group at highest in-hospital mortality [68, 69]. Subsequent analysis of the SHOCK trial along with the extensive use of invasive haemodynamic monitoring led to a more nuanced spectrum of CS haemodynamics. In this regard, up to one-third of patients with AMI-CS have signs of tissue hypoperfusion without pulmonary congestion at presentation (euvolemic CS corresponding to a “cold and dry” profile), with similar ominous prognosis [70]. Although sustained hypotension is typically required to define classic CS, there is an increasingly recognized subgroup of pre-cardiogenic shock patients who experience either isolated hypoperfusion without hypotension (due to abnormally elevated SVR) or relative hypotension without hypoperfusion, with hypoperfusion portending worse outcomes that hypotension [71]. This has led to the proposal of the concept of “normotensive CS”, which warrants timely diagnosis given its ominous prognosis—similar to that of overt CS—if unrecognized [71, 72]. Conversely, patients with isolated hypotension or with relative hypotension (>30 mmHg drop from baseline) would be labelled as “beginning” CS (i.e., SCAI stage B) [66].

Moreover, there has been growing recognition of systemic inflammatory response (SIRS) as a pathophysiological consequence of the CS cascade [73], characterized by microvascular disfunction and uncoupling of the micro- and microcirculation, leading to inappropriate peripheral profound vasodilation [74] and worsening end-organ damage. These patients may feature a “warm and wet” profile. SIRS associates with worse CS severity and higher mortality [73]. Early data from SHOCK trial revealed that SIRS may be present in up to 20% AMI-CS patients: these patients had lower SVR that those with CS without SIRS, consistent with a superimposed distributive-inflammatory phenotype, i.e., “mixed” shock (MS) [75]. Coherently, MS is reported at a rate of 20–25% of all shock admissions [1, 76]. We recently proposed a standardized definition of MS leveraging on longitudinal variations in hemodynamic and SIRS markers, where an increase in CI and/or decrease in systemic vascular resistances index (SVRi) along with increase in serum lactate and need of vasodilators downtitration/need of vasopressor support would provide the hemodynamic hallmark of ensuing vasodilatation and should be coupled with leucocytosis or leukopenia, increase in C-reactive protein, very high SvO_2_, fever or hypothermia as markers of inflammation [76]. These criteria identified a MS rate of 24.5% among patients with a primary diagnosis of CS and portended worse in-hospital prognosis. PAC helps to identify early this trajectory, providing real-time tracking of the relevant hemodynamic variables. Notably, MS criteria were met at a median time of 120 hours following CS diagnosis, implying that only extended invasive monitoring would capture these events [76].

The fourth “dry and warm” haemodynamic profile includes pure vasodilatory shock (defined by high CI, low SVR, low PAWP), and euvolemia (defined by normal CI, SVR, and PAWP).

Hemodynamic CS definitions in clinical practice guidelines and clinical trials include persistent hypotension (systolic blood pressure <90 mmHg for >30 min or need of supports to maintain SBP >90 mmHg) with low CO/CI (CI <1.8 L/min/m^2^ without support or <2.2 L/min/m^2^ with support), and normal or elevated filling pressures (i.e., central venous CVP >12–14 mmHg and PAWP >15–18 mmHg [18]. Schematically, according to invasive hemodynamics CS can be further classified as LV-dominant (low CI, high PAWP, low CVP), RV-dominant (low CI, low PAWP, high CVP) and biventricular-shock (low CI, high PAWP, high CVP). The CSWG proposed a threshold of 18 mmHg for PAWP and 12 mmHg for CVP [24]. Data for the SHOCK trial and from the CSWG demonstrated that biventricular involvement is present in 40–50% of CS patients [24, 77].

In 2019 a multidisciplinary group of experts convened by the SCAI was assembled to derive a simple and intuitive classification schema for CS including five stages of increasing shock severity labelled from A (“at risk”) to E (“extremis”) based on clinical findings, biochemical markers and haemodynamic data [78, 79]. The Authors emphasized the dynamic nature of the SCAI classification, with a patient that may start as a SCAI B stage (i.e., hypotensive without clinical and biochemical signs of hypoperfusion) and then worsen over time to a higher stage. Conversely, patients may also stabilize and improve from worse to better CS stages [79]. In this context, real-time data from invasive haemodynamic could timely inform about patient transition between stages: for example, PAC may demonstrate early—in a previously hypotensive, non-hypoperfused patient—a drop in CI, SvO_2_ and increase in PAWP, marking a transition from a SCAI B to a SCAI C CS stage).

**Fig. 4.**
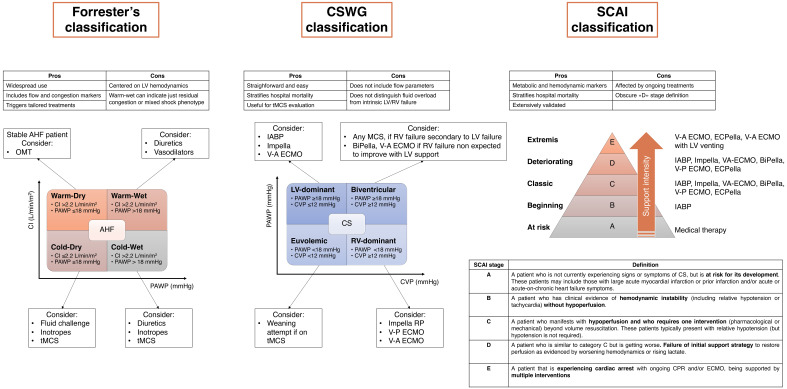
**Summary of common CS classifications and framework of supportive measures according to CS phenotypes**. AHF, acute heart failure; CPR, cardiopulmonary resuscitation; CI, cardiac index; CS, cardiogenic shock; CSWG, Cardiogenic Shock Working Group; CVP, central venous pressure; IABP, intra-aortic balloon pump; LV, left ventricular; MS, mixed shock; OMT, optimal medical therapy; PAWP, pulmonary artery wedge pressure; RV, right ventricular; SCAI, Society for Cardiovascular Angiography and Interventions; tMCS, temporary mechanical circulatory support; V-A ECMO, veno-arterial extracorporeal membrane oxygenation; V-P ECMO, veno-pulmonary extracorporeal membrane oxygenation; ECPella, ECMO combined with Impella; BiPella, biventricular support with pVAD.

Most commonly adopted CS classifications are summarized in Fig. 4.

### 4.2 Haemodynamic-Based Interventions and Hemodynamic Monitoring During tMCS

Once CS is diagnosed, the primary treatment goal is to timely restore end-organ perfusion while preventing the escalation of myocardial oxygen demand and ischemia. Despite advancements in diagnostics and therapeutics, CS remains a heterogeneous and complex clinical syndrome often managed with a “one-size-fits-all” approach, which may not consider individual variability in response to specific medical interventions. In this context, invasive hemodynamic monitoring can aid in supportive measures selection and provide objective, real-time data on the effectiveness of initial therapeutic measures, including pharmacological support with inotropes and/or vasoactive drugs, or tMCS [80, 81] (Fig. 4). According to the ventricular dysfunction profile (i.e., RV-dominant, LV-dominant or biventricular CS) [18, 66] and the clinical severity based on the SCAI stage classification and the degree of hemodynamic compromise, the proper support is selected (favouring more potent devices for more profound degrees of haemodynamic failure) [81, 82]. Degree of concomitant respiratory failure and ventricular arrhythmia are also considered to select the most appropriate tMCS, as refractory cardiac arrest due to unstable rhythms and severe respiratory compromise are amenable to veno-arterial extracorporeal membrane oxygenation (V-A ECMO) support only [81].

Once the chosen supportive measure has been started, a continuous and multimodal monitoring approach is warranted [83, 84]. The combined use of various monitoring tools, including bed-side trans-thoracic or trans-oesophageal echocardiography (TTE/TEE), biomarkers and end-organ assessment with invasive haemodynamic parameters provides the most comprehensive assessment of the cardiovascular function [84, 85]. Whereas a non-invasive hemodynamic assessment approach based on Doppler-derived estimations of pressures and flow with TTE/TEE has been shown an acceptable agreement with invasive haemodynamics [86], these measurements are episodic rather than continuous and we favour the use of PAC that supplies continuous haemodynamic data. Notably, the predictive value of hemodynamic variables and the discriminative ability of CS staging systems improve after supportive measures have started and a 24 hour re-assessment time window seems warranted to accurately track patient trajectory, highlighting the dynamic nature of CS and the impact of early care [11, 28, 87, 88]. Thus we recommend that PAC should thus be left in place for the first days of therapy and at least for the duration of tMCS [81]. During tMCS, repeated assessment of native heart function, right and left ventricular interplay and end-organ perfusion is mandatory to optimize pump flow [66, 81, 82, 83]. CO and CI provide an absolute estimate of the composite (device plus native heart) systemic flow. Parallelly, SvO_2_ can inform about the adequacy of total flow for the physiological demand of peripheral tissues [89]. Static pressures (including CVP and PAWP) confirm adequate ventricular unloading and track effective decongestion. In this framework, continuous PAC monitoring can guide further escalation/de-escalation of supports and monitor haemodynamic trends or responses to therapeutic interventions (i.e., increased pump flow/insertion of additional tMCS, titration of inotropic drugs or vasodilators, modulation of diuretic therapy). This is particularly valuable for continuous-flow devices: for example, the left-sided mAFP are highly preload-dependent, and warrant continuous assessment of the RV function and of LV filling to avoid suction events, which can cause haemolysis and/or hypotension [90, 91]. Therefore, PAWP is a key parameter to assess the degree of pulmonary venous congestion and to estimate the LVEDP and thus LV preload [92, 93], reflecting both the adequacy of LV decongestion/unloading and aiding the user to adjust the mAFP P-level or the V-A ECMO flow. Mean PAWP provides a measure of left atrium (and pulmonary) loading throughout the cardiac cycle, while end-diastolic PAWP (a-wave) provides a surrogate measure of LVEDP and—thus—of LV unloading. For example, a patient with a normal PAWP a-wave but increased mean PAWP due to giant v-wave (likely due to significant mitral regurgitation) would not benefit from increased mAFP speed or over-diuresis: indeed, these actions may excessively decrease the LV preload leading to suction events and hypotension.

Given the reappraisal of RV failure in several acute cardiac illness [77, 94], several parameters have been developed for selective evaluation of RV function. Among these, CVP to PAWP ratio is commonly used. Normally, CVP is significantly lower than PAWP; a CVP/PAWP ratio above >0.86 suggests a failing RV in the setting of AMI-CS [32, 95]. The pulmonary artery pulsatility index [PAPi = (systolic PAP – diastolic PAP)/CVP] is a reliable marker of RV function across a variety of AHF etiologies. A PAPi <0.9 in AMI-CS and <2.0 in ADHF-CS indicates significantly impaired RV function and may suggest the need for pharmacological or mechanical RV support [34, 96] (Table 2). Recently, markers of pulsatile RV afterload (i.e., pulmonary artery elastance, PaE and pulmonary artery compliance, PaC) have been shown to be powerful prognosticators in CS and in pulmonary hypertension [12, 97, 98]: these indexes combine flow and pressure measures, providing a comprehensive assessment of ventriculo-arterial coupling. Individually or in combination, these parameters can inform the user about the filling status and the degree of RV dysfunction suggesting for the need for fluid management strategies (i.e., fluid administration, diuretic therapy or renal replacement therapy) or the need for either pharmacological or mechanical RV support [94]. For example, for a patient receiving tMCS with isolated left-sided percutaneous ventricular assist device (pVAD) for biventricular CS (high CVP, high PAWP, and low CI), the evidence of a steep drop of both CVP and PAWP with a concomitant CI increase after pVAD initiation would suggest effective LV unloading with subsequent reduction in RV afterload (i.e., PAWP) and secondary RV improvement and CVP decrease. On the other hand, the evidence of a mild reduction of both PAWP and CVP after left-sided pVAD insertion and despite P-level titration would suggest residual volume overload and the need for aggressive diuretic therapy or even for renal replacement therapy (RRT) in case of inadequate decongestion; alternatively, if also lactate fail to normalize and CI and SvO_2_ do not improve this may prompt tMCS escalation to more powerful device. Moreover, in patients already on left-sided pVAD, PAC can aid to assess whether the RV is capable of keeping up with flow from the left-sided pVAD: dramatic decline in PAWP coupled with increasing CVP, low PAP and suction alarms may suggest worsening RV failure. This, in turn, may trigger supportive measures for RV failure, including inotropic titration, inhaled nitric oxide, tMCS escalation (with addition of Impella RP, veno-pulmonary ECMO or V-A ECMO). Finally, in patients receiving biventricular support with pVAD (BiPella) or with VA-ECMO combined with Impella (ECPella) the optimization of each device speed relative to the other is facilitated by PAC, as it provides essential information on PAP and PAWP and thus on ventricular interdependence.

Current guidelines also strongly recommend strict haemodynamic monitoring in patients on V-A ECMO [84]. In this setting, the thermodilution method is not suitable for CO and SvO_2_ assessment due to reduced trans-pulmonary flow leading to inaccurate readings [84]. Peripheral oxygenation may be normal despite severe pulmonary oedema due to oxygenated V-A ECMO flow, but oxygenation in the upper body may differ from that of the lower body (differential hypoxaemia). Venous blood passing through injured lungs would return deoxygenated to the LV and could be ejected in the systemic circulation. Monitoring of these patients requires oximetry preferentially from the right arm; since the retrograde V-A ECMO arterial flow mixes with the native heart flow at a variable level in the thoracic aorta (watershed level) depending on competing residual LV ejection, a reduced right arm SaO_2_ suggests desaturated blood flow from LV to the right brachio-cefalic trunk and subsequent risk of cerebral hypoxaemia. Measuring SaO_2_ from the left arm only would miss cases where the watershed is more proximal to the aortic root and desaturated blood would only flow to the right brachio-cefalic trunk. In V-A ECMO, together with bedside echocardiography, invasive hemodynamic monitoring with PAC allows for the assessment of PAWP trend, which is an indirect index of LV overload and distention. For example, once VA-ECMO has been started the evidence of increasing PAWP together with low aortic pulsatility, pulmonary congestion on chest X-ray and LV distension with minimal or no aortic valve opening on bedside echocardiography would suggest the potential need for pharmacologic (inotropes, vasodilators) or mechanical (IABP, mAFP) venting strategies [99, 100, 101].

### 4.3 Weaning and Liberation From tMCS

At present, no standardized device-specific weaning and explant protocols have been proposed or evaluated. Hence, in the absence of clinical practice guidelines, a weaning trial should be intended as a deliberate and controlled reduction in tMCS to evaluate the intrinsic native heart function’s capacity to provide the necessary circulatory support to match the body’s demands. This concept should be intended as the evidence of an improvement of native heart function sufficient to meet haemodynamic and metabolic tissues demands and not necessarily as a full restoration of pre-tMCS cardiac function. Although there are device-specific considerations for weaning and explant strategies of tMCS, criteria for readiness to wean and eventually explant include the stability of a set of clinical, hemodynamic, metabolic, echocardiographic and end-organ perfusion variables at the lowest level of tMCS [80, 102, 103]. Weaning process is usually considered after objective improvement of end-organ function and perfusion (e.g., lactate <2 mmol/L, normalized SvO_2_, improving trend of transaminases, bilirubin, INR, creatinine), adequate oxygenation and ventilation on ABG at spontaneous breathing or with minimal pressure-support ventilation (end-expiratory pressure ≤5 cmH_2_O, peak inspiratory pressure ≤15 cmH_2_O; FiO_2_ ≤0.5), minimal vasoactive drugs requirement (we usually consider a vasoactive inotropic score threshold ≤10 [104]). In addition, several hemodynamic criteria should be met prior to weaning attempt, including: a MAP >65 mmHg with good arterial pulsatility, native CI >2.2 L/min/m^2^, CVP <12 mmHg and PAWP <18 mmHg [80]. If most of the abovementioned parameters are met, a trial of tMCS weaning can be attempted, usually by reducing device support with a device-specific protocol (i.e., reducing in a stepwise fashion the pump speed to lower P-level for Impella, placing in standby mode or attempting a volume and/or ratio weaning to 1:2 or 1:3 for IABP, reducing pump speed to achieve a lower flow for V-A ECMO) [80, 102]. Moreover, the same set of haemodynamic, metabolic and echocardiographic variables should be strictly re-evaluated after any tMCS intensity step-down. On the other hand, if multiple weaning attempts fail (indicating device dependency), one should resume or escalate tMCS, addressing reversible factors, and eventually consider advanced therapies or palliative care options [80, 102].

## 5. Invasive Hemodynamic Monitoring in Advanced Heart Failure and Heart Replacement Therapies

Advanced heart failure (AdvHF) is essentially a clinical diagnosis, and several criteria have been proposed (I NEED HELP and Heart Failure Association-European Society of Cardiology (HFA-ESC) criteria): in general, these criteria leverage on the history of multiple hospitalization, loss of therapy tolerance, and end-organ damage that characterize the condition. These criteria identify a high-risk population and offer some prognostication, as overall survival is worse with increasing number of criteria met [105, 106]. Therefore, AdvHF does not need a hemodynamic diagnosis, but invasive hemodynamic monitoring is nevertheless essential in its management, especially in the following scenarios, peculiar of the AdHF cohort.

### 5.1 Heart Replacement Therapies Evaluation

AdvHF patients may be hospitalized due to ADHF-CS, and this may be a harbinger for the need of heart replacement therapies (HRT) [4, 105]. Evaluation of patient candidacy to heart transplant (HTx) or LVAD requires invasive hemodynamic evaluation. Current guidelines recommend screening for pulmonary hypertension with PAC every 3–6 months prior to HTx listing and while on HTx waitlist with the aim to rule out irreversible pulmonary hypertension [107]. In addition, in case of systolic PAP ≥50 mmHg, a trans-pulmonary gradient ≥15 mmHg, pulmonary vascular resistances >3 WU and systolic arterial pressure >85 mmHg, a vasodilatory challenge is warranted [107]. Intensive therapy with vasodilators, inotrope and/or IABP may be considered in case of persistent pulmonary hypertension despite vasodilator challenge with a repeat invasive assessment within 24–48 hours. If all the previous actions fail, implant of durable LVAD is an option [6, 107], and repeat hemodynamic profiling shall be obtained 3–6 months after implantation [107].

Similarly, invasive assessment helps to rule out significant or latent RV failure, that may complicate LVAD implantation and long-term course [108]. The following indexes denote a higher risk of post-operative right heart failure: CVP/PAWP ≥0.63 [33]; PAPi <1.85 [35] or <2.00 [36]; RVSWi ≤0.25 mmHg L/m^2^ [109] and a PaE ≥1.16 mmHg/mL [110] (Table 2).

### 5.2 After Heart Replacement Therapies

Implementation of durable HRT mandates careful patient follow-up. With the advent of new-generation fully magnetically levitated LVAD, the rate of hemocompatibility-related adverse event has significantly abated [111]. Parallelly, increasing durability of LVAD support confronts us with higher incidence of hemodynamic-related adverse events (HDRE) [112], including aortic regurgitation and RV failure: both these conditions are fostered by, but at the same time beget suboptimal hemodynamics. In addition, the RV is exquisitely sensitive to both insufficient and excessive unloading of the LV, as both pose unfavorable consequences on this chamber. Currently, despite optimized GDMT, a substantial proportion of LVAD patients shows abnormal resting hemodynamics [113] and systematic use of PAC along with echocardiography helps in achieving optimal hemodynamics [114], this holds especially true if an LVAD patient is hospitalized for AHF. Notably, achievement of optimal hemodynamics during LVAD associates with lower rates of hospitalization [115]. When LVAD is implanted in patients with pulmonary hypertension not responsive to vasodilator challenge, repeat hemodynamics shall be obtained 3–6 months after implantation to assess whether prolonged mechanical unloading has reversed the pulmonary hypertension (see above).

## 6. Invasive Hemodynamic Monitoring Implementation in Clinical Practice

As outlined, select clinical setting may warrant consideration of invasive hemodynamic monitoring over non-invasive monitoring as this may yield better phenotyping and improve clinical outcomes. These include: overt CS especially with anticipated/ongoing tMCS use [53, 60, 61, 62, 63, 64], mixed/unclear shock phenotypes [76], inconsistent findings from non-invasive assessment, and AdvAHF patients evaluated for HRT. However, implementation of invasive hemodynamic monitoring should take into account the associated costs and is subject to heterogeneous availability. Indeed, PAC is more often used at academic, tertiary centers [116], and this may depend both on variable local protocols and tool availability. From a healthcare perspective, based on hospitalizations data after 2016, use of PAC does not seem to increase—and may actually reduce—hospitalization costs when used for patients with CS [117], as opposed to the findings from older reports [118]. When compared to PiCCO system, the PAC appears less cost-effective, however, direct comparisons are difficult to obtain, as these estimates were based on old surgical and septic shock patients studies [119, 120]. Therefore, in resource-limited settings, PiCCO may provide a reasonable first-line alternative to PAC (provided reliability of the arterial waveform and no tMCS use), but the PAC—if available—hould nevertheless be considered whenever a patient fails to stabilize with the initial therapies [81].

Finally, PAC use is backed by weak recommendations from society guidelines [81] and several gaps in evidence remain open for future research, including: randomized trials demonstrating its clinical efficacy, detailed understanding of how PAC measures should inform clinical decision making, role of AI predictive algorithms in the management of the AHF/CS patient and integration of PAC data in the device-specific tMCS weaning process.

## 7. Conclusions

Invasive hemodynamic monitoring has several goals in the CICU patients and along the whole HF trajectory, as it allows for precise hemodynamic phenotyping, individualized supportive measures selection, traces the trajectory of the patient after the initial bundle of care, and prompts treatment escalation and de-escalation. In the AHF setting, systematic use of invasive hemodynamics is warranted when overt CS develops and should also be considered as an adjunctive tool in AHF patients when impending deterioration is anticipated or inconclusive findings result from non-invasive evaluation. Finally, in advanced HF invasive hemodynamic is mandatory for HRT candidacy and should be reassessed periodically as an essential tool of the patient follow-up. A wider adoption of hemodynamic monitoring can be envisioned, calling for future research to improve monitoring tools design and technology, incorporate artificial intelligence features and to inform successful clinical decision making based on hemodynamic assessment.
